# Molecular Subtypes of Pancreatic Cancer: A Review of the Literature

**DOI:** 10.3390/cimb48050502

**Published:** 2026-05-13

**Authors:** Jakub Wnuk, Wiktoria Skowron, Anna Długaszek, Joanna Sadurska, Łukasz Pietrzyński, Jacek Kabut, Iwona Gisterek-Grocholska

**Affiliations:** 1 Department of Oncology and Radiotherapy, Medical University of Silesia, 40-055 Katowice, Poland; jkb.wnuk@gmail.com (J.W.); aniakrywult@gmail.com (A.D.); lpietrzynski@gmail.com (Ł.P.); igisterek@sum.edu.pl (I.G.-G.); 2 Clinical Oncology Unit, The Professor K. Gibiński University Clinical Center of the Silesian Medical, University in Katowice, 40-055 Katowice, Poland; j.sadurska@op.pl; 3 The Student Scientific Club of the Department and Clinic of Oncology and Radiotherapy, The Faculty of Medical Sciences, Medical University of Silesia in Katowice, 40-055 Katowice, Poland; wiktoriaskowron1703@gmail.com

**Keywords:** molecular subtypes, RNA sequencing, pancreatic cancer, clinical utility

## Abstract

Pancreatic cancer (PC) is considered one of the deadliest cancers worldwide, and the number of PC-related deaths is expected to increase. Early diagnosis of PC is crucial for improving treatment outcomes. Despite improvements in overall survival (OS) in metastatic and unresectable PC due to systemic therapy, there is still a need to search for novel therapies and factors predictive of response to treatment. Cancer profiling based on genome sequencing can be used to develop targeted therapies and improve prognostics and treatment outcomes. Therefore, this review was conducted to evaluate the clinical value of molecular subtyping in pancreatic cancer as a prognostic and predictive factor in pancreatic cancer. Due to its limitations, including the lack of a registered protocol and risk of bias assessment for the included studies and those whose results were not included, this study should be considered a narrative review with a structured search strategy rather than a systematic review.

## 1. Introduction

Pancreatic ductal adenocarcinoma (PDAC) is one of the most lethal cancers worldwide. The estimated 5-year survival rate ranges from 5% to 11.5%, depending on the data source [1,2]. This is mostly due to late diagnosis and the asymptomatic course of PDAC in early stages. Pancreatic cancer (PC) was estimated to be the 12th most common cancer worldwide in 2022 (510,566 new cases; 2.6% of all cancer cases) and was responsible for 467,005 cancer-related deaths (4.8%; the sixth most common cause of cancer-related deaths worldwide) [3]. In Poland in 2022, PC was the sixth and ninth most commonly diagnosed cancer and fifth and sixth most common cause of cancer-related deaths in women and men, respectively [4]. It is estimated that by 2030, PDAC will be the second and third leading cause of cancer-related deaths in the USA and Europe, respectively [5,6,7].

PDAC remains the most common histopathological subtype of PC, accounting for about 85% of PC diagnoses. Other subtypes are rare and usually have prognostic value with little impact on treatment decision-making [8,9]. Obesity, diabetes mellitus, a diet rich in processed meat, a lack of physical activity, and tobacco smoking are the most commonly referenced risk factors of PC, along with genetic factors such as *p53*, *K-ras*, *p16*, and *PRSS1* [10,11].

In early stages, pancreatoduodenectomy is the choice of treatment in resectable cases (which are approximately 10–15% of all cases) [12]. Neoadjuvant treatment, which can either be systemic treatment with multi-drug regimens or chemoradiation, is still considered a debatable choice in cases of locally advanced pancreatic cancer (LAPC) and resectable or borderline resectable disease. Systemic adjuvant treatment with a modified FOLFIRINOX regimen is recommended for all eligible PDAC patients. In metastatic PDAC, systemic therapy with multi-drug regimens such as FOLFIRINOX or gemcitabine with nab-paclitaxel or monotherapy with gemcitabine are the preferred treatments [13]. Conroy et al. compared a FOLFIRINOX regimen to gemcitabine in monotherapy and found that the median time of overall survival (OS) was 11.1 months with FOLFIRINOX compared to 6.8 months in the gemcitabine group [14]. In a study by Sahoo et al. comparing gemcitabine accompanied by nab-paclitaxel to gemcitabine in monotherapy, OS was found to be 8.5 months in the dual-drug regimen compared to 6.7 months in the monotherapy [15].

Despite the improvements in OS in metastatic PDAC achieved with systemic therapy, its effectiveness is likely limited by the microenvironment surrounding the growing tumor. The desmoplastic response of the surrounding tissues and low angiogenesis reduce the effectiveness of chemotherapy [16]. The most recent and promising therapies, which include non-selective RAS inhibitors such as daraxonrasib, are currently undergoing clinical trials (NCT06625320). Therefore, the search for predictive factors remains necessary.

Rapid advances in the molecular pathophysiology of cancers demonstrate that even histologically similar neoplasms can harbor various molecular changes that can affect the clinical approach to patient treatment. Cancer profiling based on genome sequencing can be used to develop targeted therapies and improves prognostics and treatment outcomes [17]. Molecular subtyping for PDAC is gradually being developed; however, its clinical utility is still debatable. There have been several attempts to describe PDAC molecular subtypes. In 2011, Collisson et al. defined three molecular PDAC subtypes based on transcriptional profiling of PDAC samples and cell lines—“classical”, “quasi-mesenchymal”, and “exocrine-like”—with poorer survival for the quasi-mesenchymal subtype compared to the classical [18]. In 2016, Moffitt et al. utilized non-negative matrix factorization (NMF) to analyze gene expression in a cohort of PDAC tumors, cell lines and normal samples. They identified two tumor subtypes—a “basal-like” subtype (worse outcome) and a “classical” subtype—and two stromal subtypes: “normal” and “activated” [19]. In the same year, Bailey et al. presented their classification based on genomic analysis of recurrently mutated genes, defining four molecular subtypes—“squamous”, “pancreatic progenitor”, “immunogenic”, and “aberrantly differentiated endocrine exocrine” (ADEX)—with poor prognosis for the squamous subtype [20]. The Cancer Genome Atlas Research Network (TCGA) analysis from 2017 provided data that indicated that the proposed classifications overlap [21]. There have since been attempts to standardize the classifications [22].

Aims:

It is a valid and important task to check the clinical utility of the molecular subtyping of PDAC. The primary aim of this study was to review the current evidence for the prognostic utility of PDAC molecular profiling. The second aim was to summarize the currently available evidence on the potential association between PDAC molecular subtypes and systemic treatment outcomes.

## 2. Materials and Methods

Due to its limitations, this article should be considered a narrative review with a structured search strategy. The articles included in this review dated from 2010 to 2025. They were evaluated based on their titles and abstracts. The eligibility criteria were as follows: participants were patients with PDAC; interventions were molecular subtyping of PDAC based on gene expression analysis or ribonucleic acid (RNA) sequencing, evaluation of prognostic value by a comparison of overall survival (OS) between different subtypes, and evaluation of predictive value through comparison of the effectiveness of the systemic therapy regimens in different molecular PDAC subtypes.

PubMed and EMBASE databases were searched with the term “(pancreatic cancer or pancreatic adenocarcinoma) and (whole genome sequencing or RNA sequencing or molecular subtypes)”. We also performed a manual search of potentially eligible studies based on reference lists of extracted reviews. The search was performed from 11 to 29 August 2025. Two authors (J.W. and W.S.) performed the study screening and full-text assessment.

The extracted data included the specimen type used in molecular subtyping, the molecular subtype classification utilized in the study, the histopathological neoplasm diagnosis, neoplasm clinical stage, the prognostic utility of the classification system based on OS or other measures such as disease-specific survival (DSS), and the predictive utility of the classification based on the comparison of systemic therapy effectiveness in different subtypes.

### Limitations

This study was not initially planned as a systematic review. This study lacks a risk of bias assessment for both the included studies and the studies whose results were not included. No review protocol was created and the study was not registered. The growing interest in molecular subtyping led Robertson et al. to perform a systemic review. In their analysis, the authors focused not only on transcriptome PDAC subtyping but also on biomarker and IHC profiling [23]. In our analysis, we aimed to contribute new evidence to the predictive value research of molecular subtyping of PDAC, as demonstrated in the studies of Singh et al. [24] and Wenric et al. [25], and to distinguish between immunohistochemical surrogates for PDAC molecular subtyping. We also wanted to emphasize the ongoing efforts to understand the predictive value of PDAC molecular subtyping, as presented in the ‘Challenges in implementing molecular subtyping into clinical practice and future directions’ paragraph.

In several cases, the studies included in this analysis had overlapping populations since the authors used publicly available datasets or continued their previous studies.

The pre-analytical variability, primarily arising from the different choices of sample material and handling prior to RNA sequencing, makes it difficult to compare the included studies.

## 3. Results

**Figure 1:**
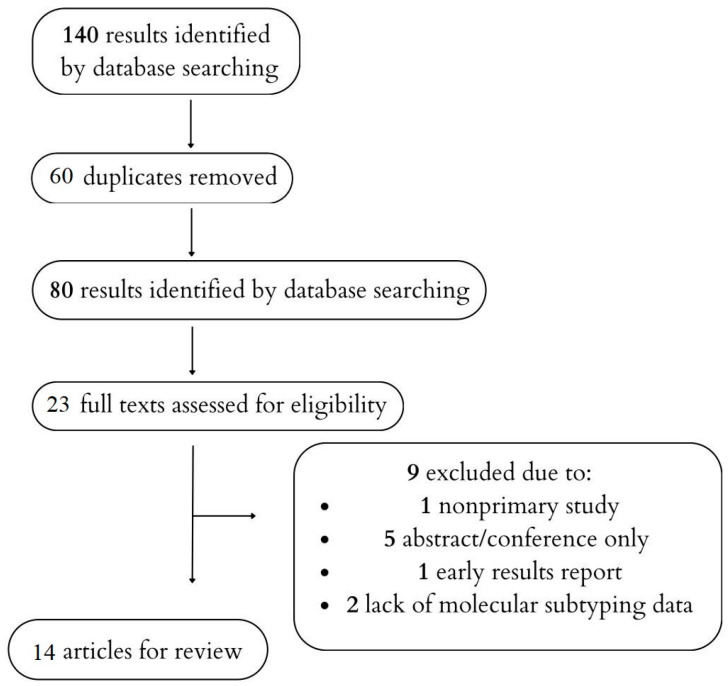
The course of the literature search.

We searched the term “(pancreatic cancer or pancreatic adenocarcinoma) and (whole genome sequencing or RNA sequencing or molecular subtypes)” and found 3681 entries in PubMed and 9741 in EMBASE. An additional seven studies were selected for further study after analyzing reviews. Following the initial search, 140 studies were selected for further analysis. After duplicate removal, 80 remained. The titles and abstracts of these studies were reviewed, and 23 were selected for full review. Out of these studies, one was excluded due to being a non-primary study, five due to being only abstracts or conference papers, one due to being an early report of results from another study for which the final results were available, and two due to a lack of data on molecular subtyping based on RNAseq or gene expression assay. Finally, 14 articles were included in the analysis [18,19,20,24,26,27,28,29,30,31,32,33,34]. The literature search approach is presented in Figure 1. The purpose of Figure 1 is illustrative rather than being a mandatory component of the PRISMA guidelines, given that this text is not a systematic review. Table 1 shows the prognostic value of the various proposed molecular subtypes of PDAC classifications. Details of the selected studies are presented in Supplementary Table S1.

### 3.1. Patient Demographics

The studies included in this review were published between 2011 and 2025. The studies comprised 6860 patients in general, of whom 6584 had survival data available. Most of the studies included patients with resectable PDAC, but five also included patients with metastatic disease [24,27,29,32,34], with two of them restricting survival analysis only to unresectable and metastatic disease [24,27].

### 3.2. Outcome Measures

Out of 14 studies included, 13 used overall survival (OS) as an outcome measure. Other measures included DSS (disease-specific survival) as the only means of measure in one study [26] and DFS (disease-free survival)in three cases [29,31,35].

### 3.3. Material Used in Studies

Out of 14 studies, 4 used freshly frozen tumor tissue (FFTT) for their analysis [26,30,33,34], 5 used formalin-fixed paraffin-embedded (FFPE) tissue [19,24,31,32,35], 2 mentioned only that the material came from PDAC resection [20,29], 1 used resected material that underwent microdissection [18], 1 used biopsied material that underwent microdissection [27], and 1 used material from both resected PDAC tissue and resected PDAC tissue that underwent microdissection [28].

### 3.4. Techniques Used to Determine Molecular Subtypes

Studies included in this analysis described molecular subtyping based on either RNA sequencing [19,20,26,27,30,31,32,33,34,35] or whole-gene expression [18,26,28,29]. One study mentioned using the PurIST algorithm [36] based on RNA sequencing [24].

### 3.5. Classification Used

Two studies used the classification presented in Bailey et al. [20,32], two used the classification from Collisson et al. [18,27], and seven used Moffitt classification (either tumor or stromal classification) [19,28,29,30,31,33,34] with the modified stromal classification of Maurer et al. [33] One study [24] used its own five-subtype classification, and one study [28] compared Bailey, Moffitt, and Collisson classifications using publicly available datasets. A study by Zhao et al. used a six-subtype classification for tumors and stroma [35]. The details of the classifications used in the studies are presented in Table 2.

### 3.6. Prognostic Utility

#### 3.6.1. Overall Survival

In Collisson et al., the classical subtype tumors presented better OS than the quasi-mesenchymal subtype in a univariate analysis (*p* = 0.038), and the molecular subtype remained an independent predictor of OS in Cox multivariate analysis (*p* = 0.024) [18]. In Jankys et al. [29] (which referenced Collisson classification), no statistically significant difference in OS was observed between the k2.cl1 (classical subtype) and k2.cl2 subtype, nor between the three subtypes separately (*p*  =  0.193) in univariate analysis. This study, however, observed differences in DFS, which we will discuss in the next section.

In Moffitt et al., patients with basal-like subtype tumors had worse median OS (11 months) compared to patients with classical subtype tumors (19 months) (*p* = 0.007). Moffitt classification also distinguished stromal subtypes. Patients with an activated stroma subtype a had worse median survival of 15 months compared to patients with a normal stroma subtype (median of 24 months) [19]. Similar results were presented in Roa-Peña et al. (worse prognosis for the basal-like subtype, HR 1.799, *p* = 0.0094) [32], Maurer et al. (worse prognosis for the basal-like epithelial subtype compared to the classical subtype, based on Kaplan–Meier survival plots) [33], Suurmejier et al. (worse OS in the basal-like compared to the classical subtype: 11 versus 16 months, *p* = 0.035; confirmed by Cox’s multivariate analysis) [30], Singh et al. (the strong basal subtype in metastatic PDAC had significantly worse OS compared to patients with the strong classical subtype in metastatic PDAC: median of 5.7 [95% CI: 5.3–6.1] vs. 10.0 [95% CI: 9.3–10.6] months, respectively) [24], and Knox et al. (the basal-like subtype had worse prognosis in Cox’s multivariate analysis: HR 2.213 (CI 1.522–3.217), *p* < 0.001) [27].

In Bailey et al., the squamous subtype of PDAC had significantly worse OS compared to other subtypes (13.3 months), which was confirmed in multivariate analysis (HR: 2.23 (1.23–4.07); *p* = 0.0086) [20]. Similar results were presented by Dreyer who used DSS to measure prognostic utility, which are discussed further below [26].

Puleo et al., who present results for five PDAC molecular subtypes (basal-like, stroma-activated, desmoplastic, pure classical, and immune classical), confirmed the worst OS for the basal-like subtype (median OS of 10.3 months), while the pure classical and the immune classical subtypes showed an equivalently good prognosis (median OS values of 43.1 and 37.4 months, respectively). The five-subtype model was significantly associated with survival in both univariate (OS log-rank test, *p* = 4 × 10^−9^) and multivariate analyses. The study also used DFS as the measure of prognostic value, and observations for OS were similar in the case of DFS [31].

Birnbaum et al., who compared three different classifications in one study (Moffitt, Bailey, and Collisson), confirmed the utility of Moffitt’s “tumor” classification (*p* = 1.56 × 10^−3^) and Bailey classification (*p* = 1.69 × 10^−2^) in multivariate survival analysis, while finding that Collisson classification did not have any prognostic value [28].

Similar results were presented by O’Kane et al., where the classical subtype was associated with better OS compared to the basal subtype (OS 9.3 months vs. 5.9 months) [34].

#### 3.6.2. Disease-Specific Survival

In Dreyer et al., the squamous subtype of PDAC was associated with worse DSS compared to other subtypes in univariate analysis (median survival 14.9 vs. 26.5 months, *p* < 0.001), which was confirmed by multivariate analysis (squamous molecular subtype was an independent risk factor for worse DSS (HR, 1.54; 95% CI, 1.04–2.28, *p* = 0.032) [26].

#### 3.6.3. Disease-Free Survival

In Jankys et al. [29] (which used Collisson classification), univariate analysis found DFS to be significantly better for k2.cl1 (classical subtype) than for k2.cl2 (exocrine-like subtype and quasi-mesenchymal subtype) (*p*  =  0.035); it was also better for k3.cl1 (classical subtype) than k3.cl2 (exocrine-like subtype) (*p*  =  0.026).

In Puleo et al., the worst DFS was associated with the basal-like subtype (4.57 months), while the classical subtype had the best DFS (20.13 months) (log rank 2 × 10^−7^) [31].

In Zhao et al., the pure basal-like subtype and the stroma-activated subtype were associated with worse DFS (medians of 10.23 months and 11.74 months, respectively). Similar data were noted for OS, but were only available in the form of a figure [35].

### 3.7. Predictive Value in Different Molecular Subtypes

Five of the included studies directly referenced systemic therapy and its effectiveness in the context of molecular subtypes of PDAC. Table 3 presents the results of systemic treatment for different molecular subtypes. Details of the included studies can be found in Supplementary Table S2. However, these results remain preliminary.

## 4. Discussion

Over the years, different molecular subtype classifications based on the transcriptome (RNA profiling) have been proposed. First, in 2011, Collisson et al. used hybridization array-based RNA expression methods to define three subtypes of PDAC: classical (with high expression of adhesion-associated and epithelial genes), quasi-mesenchymal (with high expression of mesenchyme-associated genes), and exocrine-like (with high expression of tumor cell-derived digestive enzyme genes, supported by immunohistochemical staining) [18].

Moffitt et al. [19] derived a classification system distinguishing between two main cell components present in the specimen: the tumor and stromal components. For tumor classification, they defined classical and basal-like subtypes of PDAC, while for stromal classification, they defined activated (genes associated with macrophages, genes associated with tumor promotion: secreted protein SPARC, WNT family members, gelatinase B (MMP9), stromelysin 3 (MMP11), and fibroblast activation protein (FAP)) and normal (with high expression of pancreatic stellate cell markers: smooth muscle actin, vimentin, and desmin) subtypes. They also noticed that Collisson et al.’s subtypes overlapped with their classification. Collisson’s exocrine-like subtype had expression indistinguishable from normal pancreatic tissue, the quasi-mesenchymal subtype had features of the basal-like subtype and the stromal subtypes, while the classical subtypes overlapped in both classifications [19].

Bailey classification described four subtypes: squamous, pancreatic progenitor, immunogenic, and aberrantly differentiated endocrine exocrine (ADEX). These subtypes were also associated with histological characteristics. Three out of four overlapped directly with Collisson classification: quasi-mesenchymal and squamous; classical and pancreatic progenitor; exocrine-like and ADEX. The immunogenic subtype was associated with increased immune infiltration [20].

The Cancer Genome Atlas Research Network attempted to summarize and unify the overlapping classifications. Using the computational ABSOLUTE algorithm [37], they divided their cohort into high-purity (≥33%) and low-purity (<33%) groups to address the challenge of low tumor cellularity of samples. Their observations led to the conclusion that in high-purity samples, PDAC can be classified into a basal-like/squamous group and a classical/progenitor group. Immunogenic and ADEX or exocrine-like subtypes were associated with low purity samples [21].

Puelo et al. [31] addressed the problem of variable neoplastic cell levels in tumor samples and the influence of stromal cells on the tumor microenvironment by proposing a redefined classification with five molecular subtypes of PDAC: (1) pure classic (with a low stromal signal), (2) immune classic (with a stronger stromal signal), (3) stroma-activated (a basal subtype enriched with a stroma component), (4) desmoplastic (a low tumoral component with strong stromal activation), and (5) pure basal-like (with a low stromal signal). The first two could be referred to as classical subtypes in the previous classifications, while the latter three are composed of classical and basal-like subtypes. The tumor compartment of PDAC can be described by two subtypes (basal-like and classical), as suggested in previous studies.

In 2019, Collisson et al. took a harmonized approach to classifying molecular PDAC subtypes, dividing them into two main lineages created by the loss of endodermal–pancreatic identity: squamous and classical–pancreatic. Both subtypes can be further divided into classes [22]. One of the challenges of tumor analysis is the fact that gene expression is averaged between tumor and stromal compartments. Thus, ensuring the use of high-purity material remains crucial to the results. Differences in the material used in the chosen studies were also significant. In the first study describing molecular subtypes, to ensure a high number of PDAC cells in the tested material and to decrease the number of stromal cells and normal exocrine pancreatic cells causing interference, the epithelial cells in the samples were physically microdissected [18]. In the study by Moffitt et al. from 2015, the problem of varied gene expression in stromal and tumor compartments was addressed by using the non-negative matrix factorization (NMF) technique to perform virtual microdissection [19], which has previously been reported to be useful in other human neoplasms [38,39]. In Bailey classification from 2016, based on RNA sequencing (RNAseq), the material used in the analysis was derived from bulk resected PDAC tumors and metastases with a high epithelial content (>40% cellularity) to balance stromal gene expression [20]. The summary of similarities and differences between main classifiers are presented in Table 4.

Despite the low gene-level overlap across the signature gene lists, the prognostic findings are remarkably concordant. Birnbaum et al. applied all four classifiers (Collisson, Moffitt tumor, Moffitt stroma, and Bailey) to a pooled series of 846 PDAC samples from 15 public datasets and demonstrated a concordance rate of 73% to 86% when assigning “good prognosis” or “poor prognosis” between the three tumor epithelium-based classifiers (Collisson, Moffitt tumor, Bailey). The concordance was substantially lower (50–60%) when comparing tumor-based classifiers with the Moffitt stroma classifier, reflecting the complementary biological information captured by stromal gene lists. Cramer’s V analysis showed the strongest agreement between Bailey classification and the Moffitt tumor classifier. Multivariate survival analysis incorporating all four classifiers simultaneously retained only Bailey classification and the Moffitt stroma classification as independently prognostic, highlighting their complementarity [28].

In the TCGA study, when only high-purity samples were considered, Bailey’s squamous samples showed significant overlap with Moffitt’s basal-like samples, while Bailey’s pancreatic progenitor samples and Collisson’s classical samples largely overlapped with Moffitt’s classical samples [21].

The main PDAC molecular classification systems converge on a fundamental dichotomy between a classical/pancreatic progenitor lineage—associated with preserved endodermal identity and a more favorable prognosis—and a basal-like/squamous lineage—characterized by a loss of differentiation, EMT enrichment, and a poorer clinical outcome. Additional subtypes identified in earlier studies (ADEX, exocrine-like, immunogenic) likely reflect stromal, immune or normal tissue contamination and are not reliably reproduced in high-purity or deconvoluted analyses. The high but imperfect concordance between classifiers, driven in part by intratumoral heterogeneity and technical variability, shows the need for standardized, clinically validated subtyping tools and prospective evaluation of their impact on treatment decision-making.

Taken together, despite differences in methodology, nomenclature, and sample composition, the major transcriptomic classification systems of PDAC converge on a shared biological framework. In practice, most tumors can be consistently mapped onto two principal epithelial lineages: a classical/pancreatic progenitor group, associated with preserved differentiation and more favorable outcomes, and a basal-like/squamous group, characterized by a loss of epithelial identity and poorer prognosis.

In retrospect, while preparing this review, it became increasingly clear to us that many of the additional subtypes described across studies—such as ADEX, immunogenic, or exocrine-like—are less consistently reproduced and are often influenced by stromal, immune, or low-purity sample components rather than representing stable, tumor-intrinsic categories. This perspective does not invalidate earlier classifications, but rather places them within a more unified and biologically coherent framework.

Framing the literature in this way may help reconcile some of the apparent inconsistencies between studies and provides a clearer conceptual basis for interpreting the clinical relevance of molecular subtyping in PDAC.

### 4.1. Methodological Differences

The classification systems differ not only in nomenclature but also in the technologies and analytical approaches used. Collisson et al. [18] employed global gene expression microarray analysis on surgically microdissected epithelial tumor samples and developed a 62-gene PDA signature. Moffitt et al. used microarray data in combination with non-negative matrix factorization (NMF), a form of “virtual microdissection”, to computationally separate tumor-specific and stroma-specific gene expression programs from bulk tumor specimens, thereby defining both tumor subtypes (classical and basal-like) and stromal subtypes (normal and activated) [19]. Bailey et al. combined whole-genome sequencing with RNA sequencing of 456 resected specimens enriched for high epithelial content (≥40% neoplastic cellularity) and identified four subtypes through unsupervised clustering of gene programs [20]. The Cancer Genome Atlas Research Network (TCGA) subsequently performed integrated multi-platform profiling—including whole-exome sequencing, RNAseq, miRNAseq, DNA methylation arrays and reverse-phase protein arrays (RPPAs)—on 150 PDAC specimens that included tumors with characteristically low neoplastic cellularity (median ABSOLUTE purity 33%) [21].

The transcriptome consists of the complete set of RNAs produced by a cell. Changes in RNA expression levels are caused by different internal and external factors, including developmental signals and environmental stress.

One method of testing gene expression is bulk RNA sequencing. This method utilizes the NGS (next-generation sequencing) technique to measure levels of RNA expression. However, this method can only provide information about the average expression profile of a heterogeneous cell population since it relies on a bulk tissue sample [40].

Due to the infiltration of PDAC into the surrounding parenchyma, bulk tissue samples may contain non-neoplastic, atrophic, or metaplastic cells, which can interfere with RNA sequencing levels. One solution to this problem is laser capture microdissection (LCM), which allows for the isolation of a compartment specific to the tumor epithelium, or macrodissection. LCM employs special staining and cutting of formalin-fixed, paraffin-embedded tumor specimens into 10-micrometer slices to ensure the separation of tumor and stromal cells [41].

“Virtual” microdissection is another approach to separating tumor compartments with different molecular signatures. It uses the non-negative matrix factorization (NMF) data mining technique to identify modules in biological networks [42]. In the case of PDAC, it has enabled virtual distinction between neoplastic and surrounding non-neoplastic tissue. This method has been applied not only to PDAC but also to other human cancer samples [38,43]. However, the interaction effects between the identified modules can overlap, thus prompting the proposal of other methods to improve this technique, such as NMFNA [44].

### 4.2. Material Source

The reliability of study comparisons is impacted by different tissue sources. While FFPE samples are most commonly used in clinical practice due to their stability and low storage costs, they may have low tumor cellularity, which can affect molecular testing. In contrast, fresh-frozen or laser-captured microdissected samples provide higher-quality DNA/RNA but are less practical for routine use, complicating the implementation and comparability of molecular subtyping [20,45,46].

In a study by Gao et al., the researchers compared fresh-frozen tissue with FFPE tissue undergoing multi-gene profiling in colorectal cancer patients. The results were highly concordant at the variant and gene levels, with some important differences. The authors suggested that FFPE should not routinely replace fresh-frozen tissue, which is considered a reasonable alternative [47].

Purity-aware analyses have shown that, when restricted to high-purity specimens, PDAC tumors consistently segregate into two epithelial lineages (classical/pancreatic–progenitor vs. basal-like/squamous), while additional subtypes become strongly enriched in low-purity samples and largely reflect non-neoplastic components. These differences in sample type, pre-analytic processing, and purity introduce important limitations when comparing results across cohorts and likely explain part of the inconsistent prognostic strength reported for some classifications. Therefore, tumor purity and sampling strategy must be considered when interpreting subtype calls, and low-purity RNA-seq datasets should be viewed in light of the increased risk of misclassification, especially if purity-independent or deconvolution tools are not applied [21].

Importantly, these methodological and sample-related differences are not merely technical considerations but have direct implications for the interpretation of the available evidence. In retrospect, while analyzing the included studies, it became increasingly apparent to us that at least part of the variability in reported prognostic and predictive effects may stem from differences in tumor purity, stromal admixture, and sample processing rather than true biological discrepancies between cohorts. For instance, classifications derived from bulk RNA sequencing of low-purity samples may partially reflect stromal or immune components, which can lead to apparent subtype distinctions that are not entirely tumor-intrinsic.

This may help explain why some studies report weaker or inconsistent associations between molecular subtypes and survival outcomes, despite an overall trend favoring the classical versus basal-like dichotomy. In other words, the heterogeneity observed across studies should not be interpreted solely as a lack of robustness of molecular subtyping itself, but rather as a consequence of differences in how the underlying biological signal is captured.

At the same time, this reinforces one of the central limitations of the current literature: without standardized approaches to sample selection, purity assessment, and data processing, direct comparison between studies remains challenging. Therefore, methodological variability and sample composition should be explicitly considered when interpreting subtype classifications, and future research should aim to integrate purity-aware or deconvolution-based approaches more consistently.

### 4.3. Prognostic Utility of Molecular Subtyping of PDAC

The studies included in this analysis consistently suggest that quasi-mesenchymal, squamous, or basal-like subtypes of PDAC are associated with poorer prognosis based on OS, DFS, or DSS. However, while the direction of this association appears relatively stable across studies, its strength and statistical robustness are not uniform. In particular, not all classification systems retain independent prognostic value in multivariate analyses, and their performance may vary depending on study design, patient population, and methodological factors.

Birnbaum et al. compared different molecular subtypes based on publicly available data. According to their study, threeof the tested classifications (Moffitt, Bailey, and Collisson) had statistically significant results in univariate analysis. However, only Moffitt and Bailey classifications remained significant in multivariate analysis, and Collisson classification was found to have no prognostic value [28].

Zhao et al. compared the prognostic utility of the six previously described molecular subtypes [31] and their transcriptomic components, and found that the components showed better performance in predicting DFS and OS for the patients included in their study [35]. Based on this, they formed a prognostic model, including clinic pathological factors such as lymph node assessment and surgical margin status, which resulted in a model significantly associated with DFS [35].

Taken together, these findings suggest that although multiple classification systems capture a similar underlying biological signal, they differ in their reproducibility and prognostic performance. Among the commonly used classifiers, those proposed by Moffitt and Bailey appear to demonstrate more consistent prognostic value across studies and are more likely to retain significance in multivariate analyses. In contrast, other systems, such as the Collisson classification, show greater variability in their prognostic performance, which may reflect differences in methodology, sample composition, or cohort characteristics.

### 4.4. Predictive Utility of Molecular Subtyping in PDAC

A growing but still limited body of evidence suggests that PDAC molecular subtypes may have predictive value for systemic therapy. Although several studies report consistent differences in treatment response between classical and basal-like tumors, the available data remain relatively sparse and are derived largely from retrospective analyses, with only a small number of studies directly addressing treatment outcomes in this context. Therefore, these findings should currently be interpreted as emerging evidence rather than as a basis for routine clinical decision-making.

Classical/pancreatic–progenitor tumors appear to be better treated by intensified regimens such as modified FOLFIRINOX, whereas basal-like/squamous tumors have consistently poorer outcomes and seem relatively chemoresistant across both FOLFIRINOX and gemcitabine plus nab-paclitaxel. This pattern highlights the need for further studies to evaluate the potential role of molecular subtyping in identifying patients who are more likely to benefit from aggressive combination chemotherapy, as well as recognizing those for whom escalation of standard cytotoxics may offer limited additional benefit. Basal-like tumors appear to show biological features associated with treatment resistance, including a loss of pancreatic lineage specifiers, enrichment of EMT and stem-like programs, and activation of MYC/ΔNp63-driven signaling, which provides a plausible mechanistic basis for their inferior responses.

Preclinical studies by Collisson et al. provided preliminary evidence of differences in drug sensitivity among subtypes. On average, cell lines with a QM-PDA (quasi-mesenchymal/basal-like) phenotype were more sensitive to gemcitabine, while erlotinib was more effective against the classical subtype. These results support the relevance of distinct signaling pathway activity between the subtypes, including classical PDAC’s greater dependence on EGFR signaling [18].

In Dreyer’s analysis, the squamous/basal-like subtype had a notably less favorable clinical course. These patients were less likely to qualify for adjuvant treatment, and their prognosis was significantly worse, regardless of the therapy applied. This suggests a biologically based resistance to standard regimens, such as FOLFIRINOX or gemcitabine, used in chemotherapy. Conversely, the classical subtype was characterized by greater chemosensitivity, consistent with previous observations that tumors with a dominant glandular phenotype respond better to fluoropyrimidines and platinum derivatives [26].

The data from Knox et al. concerning patients with unresectable, largely metastatic PDAC may indicate that tumors with a basal-like phenotype respond particularly poorly to modified FOLFIRINOX. The median survival of 6.5 months is significantly shorter than that observed in population analyses of tumors with a classical phenotype. These findings likely indicate the aggressive nature of the basal-like subtype and its limited responsiveness to intensified chemotherapy [27].

After completing the literature review for this paper, a study by Wenric et al. [25] was published presenting real-world data evaluating PurIST classifier terms for therapy selection in PDAC. The study confirmed that the basal subtype had a poorer prognosis compared to the classical subtype (OS of 7 months vs. 11.8 months, respectively). The study also provided data showing that in the case of the classical subtype, the FOLFIRINOX regimen might be associated with better relative risk reduction in death than the gemcitabine + nab-paclitaxel regimen. There was no comparable risk reduction in basal subtype patients [25].

The basal-like subtype is considered chemoresistant based on the results of the studies mentioned above [18,21,27,34,48]. A study by Martinelli et al. provided data indicating that a loss of GATA6 expression (which appears to be associated with the basal-like subtype of PDAC) might be related to chemoresistance. Silencing of GATA6 was associated with downregulation of E-cadherin and upregulation of vimentin, leading to increased cell scattering in colonies and convergence towards epithelial–mesenchymal transition (EMT), which may be associated with chemoresistance and increased metastatic dissemination in PDAC [49,50]. Conversely, GATA6 overexpression led to the formation of more compact colonies, demonstrating that GATA6 is responsible for the epithelial phenotype in PDAC [51]. Cells with a loss of GATA6 expression were also more likely to invade in vitro and were associated with a lower response to 5-fluorouracil, as analyzed using xenograft cell lines (TKCC) and patients recruited in the ESPAC-3 trial [52]. This effect was not observed in relation to gemcitabine or paclitaxel. Furthermore, knocking down GATA6 in TKCC cell lines with high GATA6 expression and high 5-FU sensitivity did not decrease their drug sensitivity. This has led researchers to conclude that GATA6 is part of a molecular phenotype involved in drug response, but is not its major driver [51].

In the study by Singh et al., the microenvironment of strongly basal-like tumors expressed a significantly higher IFNγ signature than strongly classical tumors, with high PD-L1 expression. The authors hypothesize that this phenomenon may be related to a good response to immune checkpoint inhibitors [53,54].

In this context, the Purity-Independent Subtyping of Tumors (PurIST) classifier is a clinically attractive, purity-independent tool that assigns tumors to classical or basal-like categories using a limited gene set. It has been analytically validated on routine FFPE material, with real-world data confirming worse survival in basal tumors and suggesting preferential benefit from FOLFIRINOX in classical cases; however, prospective biomarker-stratified trials are still required before confirming its routine predictive use [36,55].

### 4.5. Challenges in Implementing Molecular Subtyping into Clinical Practice and Future Directions

RNAseq-based molecular classification of PDAC has not yet entered routine clinical practice, mainly because it requires costly next-generation sequencing, specialized bioinformatics, standardized pre-analytic handling, and sufficient high-quality tissue, which are not universally available. In addition, differences between platforms, gene panels, analytic pipelines, and cut-offs complicate harmonization across institutions and limit the development of widely accepted clinical algorithms.

Another challenge is associated with material source for molecular testing, as described above in previous paragraphs. In routine practice, most of diagnostic material for PDAC comes from fine-needle biopsy, resulting in a small amount of collectable material, which prevents the effective collection of high-cellularity material [56].

Another challenge in the clinical implementation of molecular classification using RNAseq is the time required for analysis, which typically lasts several weeks, given the rapid progression of pancreatic adenocarcinoma. Future research should focus on methods that enable faster diagnosis.

To improve feasibility, immunohistochemical surrogates such as GATA6 (classical/pancreatic–progenitor) and KRT17 or related basal keratins (basal-like/squamous) have been proposed as lower-cost alternatives; however, they still suffer from variability in staining and scoring, lack of consensus thresholds, and limited prospective validation, so they remain complementary rather than definitive substitutes for transcriptomic subtyping [57,58]. Therefore, these surrogates remain feasible for further research.

O’Kane et al. describe attempts to reduce the cost of molecular subtyping, and correlated the Moffitt classifier and GATA6 expression using an in situ hybridization assay (ISH). A higher semiquantitative score for GATA6 expression was present in the classical subtype. To facilitate clinical application, GATA6 expression was also examined using immunohistochemical diagnostic tests (IHCs) with an anti-GATA6 antibody, which correlated with ISH. However, the number of samples available for comparison was relatively low in this study, and further analysis is required. Furthermore, post hoc analysis of this study revealed that keratin 5 (CK5) exhibited the most significant expression differences between basal-like and classical tumors, complementing GATA6 expression [34].

**Figure 2:**
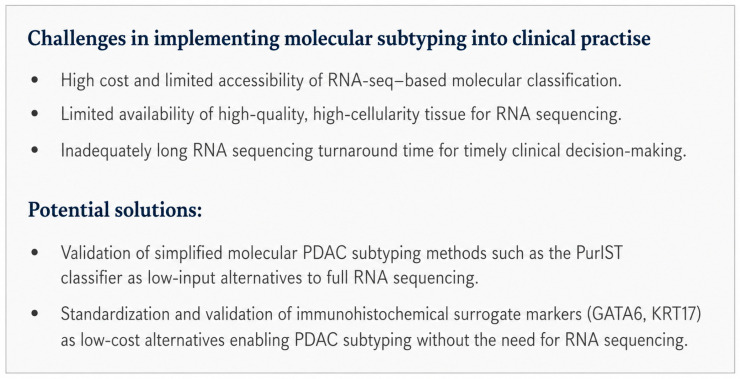
Key clinical challenges with PDAC molecular subtyping implementation into clinical practice.

A further critical limitation relates to the lack of consensus regarding the optimal timing of molecular profiling. In many studies, subtyping is performed retrospectively or on post-operative specimens, which restricts its utility to prognostic assessment rather than treatment guidance. Only a small number of prospective trials have incorporated baseline profiling prior to treatment initiation, and data directly comparing different time points of assessment are scarce. Consequently, it remains unclear at which stage of the disease course molecular subtyping should be performed to achieve maximal clinical benefit. The ongoing clinical trials (the PANCREAS study and the NCT05314998 trial) are planned to address these questions. Figure 2 presents a summary of the challenges that need to be overcome before molecular subtyping can be implemented into clinical practice. The PANCREAS study aims to examine the choice of adjuvant/neoadjuvant treatment in the context of a molecular subtype based on the PurIST classifier (NCT04683315). The NCT05314998 study will investigate the choice of adjuvant treatment in resected PDAC. The plan is to use either gemcitabine or oxaliplatin-based regimens. The decision will be made based on standard clinical criteria or according to a transcriptomic treatment-specific stratification signature. The PASS-01 study will include an evaluation of molecular subtypes in relation to treatment choice in patients with untreated metastatic pancreatic ductal adenocarcinoma (NCT04469556).

## 5. Conclusions

Molecular profiling of PDAC is a developing technique for which there is growing evidence of clinical utility as a prognostic marker. Despite their methodological variability, a consistent pattern emerges across the reviewed studies, with most classification systems converging on a fundamental dichotomy between classical and basal-like tumor lineages. Of the selected studies, the three most commonly used PDAC molecular subtype classifiers were the Moffitt, Bailey, and Collisson classifications, which had the strongest evidence of prognostic utility. However, differences in the technologies and analytical approaches used in the various classifications mean that further research on this topic is required.

The predictive value of molecular subtyping in PDAC remains to be fully described, with clinical trials in progress. Nevertheless, a worse response to chemotherapy has been observed in cases of the basal-like and squamous subtypes. As only five studies on this topic were included in this analysis, the predictive value of PDAC molecular subtyping requires further research.

Both the lack of unified nomenclature and costs hinder the clinical implementation of molecular subtyping into clinical practice. The development of methods for molecular subtyping should focus on the use of easily accessible clinical materials, like FFPE tissues, and reducing the testing time and cost.

Analysis of the material included in this review and the developmental directions of molecular subtyping of PDAC suggests that it is moving towards the use of more readily available materials (such as FFPE tissue samples, including biopsy samples) and analytical methods based on data mining techniques (such as those used in the Moffitt classification and the PurIST algorithm). This could reduce the time required to obtain results and the cost of the procedure.

However, due to the limitations mentioned earlier in this article, we would like to emphasize that this review should be considered a narrative review with a structured search strategy. Therefore, the strength of the recommended directions of developing PDAC molecular subtyping and the conclusions is diminished.

## Figures and Tables

**Table 1:** Prognostic utility of PDAC molecular subtypes in selected studies (OS—overall survival, QM-PDA—quasi-mesenchymal pancreatic ductal adenocarcinoma, FFPE—formalin-fixed paraffin-embedded, NMF—non-negative matrix factorization, DFS—disease-free survival, DSS—disease-specific survival).

Study	Samples Analyzed	Molecular Subtyping Method	Clinical Stage	Prognostic Outcome Measure	Prognostic Outcome
Collisson et al. [18]	Resected tumor material, microdissected	Global gene expression analysis	Resectable tumor, celllines	OS	Classical subtype—better OS
Moffitt et al. [19]	FFPE (NMF clustering)	RNA sequencing	Resectable tumor	OS	Classical subtype—better OS Normal stroma subtype—better OS
Bailey et al. [20]	Resected tumor material	RNA sequencing	Resectable tumor	OS	Squamous subtype—worse OS
Janky et al. [29]	Resected tumor material (NMF clustering)	Gene expression profiles	Resectable tumor, metastatic PDAC	DFS and OS	DFS better for k2.cl1 compared to k2.cl2 DFS better for k3.cl1compared to k3.cl2
Birnbaum et al. [28]	Resected tumor material, microdissected	Gene expression profiles	Resectable tumor	OS	Moffitt: classical subtype—better OS Bailey: squamous subtype—worse OS Collisson: quasi-mesenchymal—worse OS
Puleo et al. [31]	FFPE	RNA sequencing	Resectable tumor	DFS and OS	Basal-like subtype—the worst outcome Pure classical and immune classical subtypes—equivalently good outcomes
Roa-Peña et al. [32]	FFPE	RNA sequencing	Resectable tumor, metastatic PDAC	OS	Basal-like subtype—worse OS
Maurer et al. [33]	Fresh-frozen tumor tissue	RNA sequencing	Resectable tumor	OS	Basal-like—worse OS Stromal subtypes—better prognosis than ECM-rich tumors
O’Kane et al. [34]	Fresh-frozen tumor tissue which undergoes laser capture microdissection	RNA sequencing	Resectable tumor, metastatic tumor	OS	Basal like subtype—worse OS
Suurmeijer et al. [30]	Fresh-frozen tumor tissue	RNA sequencing	Resectable tumor	OS	Basal-like subtype—worse OS
Dreyer et al. [26]	Fresh-frozen tumor tissue	RNA sequencing and microarray gene expression	Resectable tumor	DSS	Squamous subtype—worse OS
Singh et al. [24]	FFPE	RNA sequencing	Metastatic PDAC	OS	Strongly basal-like subtype—worse OS
Knox et al. [27]	Biopsy after laser capture microdissection	RNA sequencing	Unresectable tumor, metastatic PDAC	OS	Basal like subtype—worse OS
Zhao et al. [35]	FFPE	RNA sequencing and microarray gene expression	Resectable PDAC	DFS and OS	Pure basal-like subtype and stroma-activated subtype—worse DFS

**Table 2:** Molecular subtype classifications used in selected studies.

Study	Year	Molecular Classification	Tumor Subtypes	Stromal Subtypes
Collisson et al. [18]	2011	Collisson classification	Classical	-
			Quasi-mesenchymal	
			Exocrine-like	
Moffitt et al. [19]	2015	Moffitt classification	Classical	Normal
			Basal-like	Activated
Bailey et al. [20]	2016	Bailey classification	Squamous	-
			Progenitor	
			ADEX	
			Immunogenic	
Janky et al. [29]	2016	Corresponding to Collisson classification	2- and 3-subtype classification	-
Birnbaum et al. [28]	2017	Collisson, Moffitt, and Bailey classifications	Comparison of 3 different classifications	-
Puleo et al. [31]	2018	Puleo classification	Pure basal-like	
			Stroma-activated	
			Desmoplastic	
			Pure classical	
			Immune classical	
Roa-Peña et al. [32]	2019	Moffitt classification	Classical	--
			Basal-like	
Maurer et al. [33]	2019	Moffitt classification and own stromal classification	Classical	Immune-rich
			Basal-like	Extracellular matrix-associated pathways (ECM-rich)
Suurmeijer et al. [30]	2022	Moffitt classification based on PurIST algorithm	Classical	-
			Basal-like	
Dreyer et al. [26]	2023	Bailey classification	Classical (including ADEX and immunogenic subtype)	-
			Squamous	
Singh et al. [24]	2024	Moffitt classification based on PurIST algorithm	Classical	-
			Basal-like	
Knox et al. [27]	2025	Moffitt classification	Classical	-
			Basal-like	
Zhao et al. [35]	2021	Based on Puleo classification	Classical	Inactive structural stroma
				Activated stroma
			Basal-like	Inflammatory stroma
				Immune stroma
O’Kane et al. [34]	2019	Moffitt classification	Classical	--
			Basal-like	

**Table 3:** Molecular subtypes and their influence on treatment outcomes (NAT—neoadjuvant treatment, GemCAP—gemcitabine-capecitabine, mFFX—modified FOLFIRINOX, FFX—FOLFIRINOX).

Study	Year	Treatment (Adjuvant/Neoadjuvant/Palliative)	Regimens	Treatment Outcomes
Dreyer et al. [26]	2023	NAT and adjuvant	- mFFX - Gemcitabine - Chemoradiation with GemCAP	Squamous subtype—less likely to receive adjuvant therapy; worse prognosis
Collisson et al. [18]	2011	Cell lines (in vitro)	- Erlotinib - Gemcitabine	QM-PDA subtype—sensitive to gemcitabine Classical subtype—sensitive to erlotinib
Knox et al. [27]	2025	Palliative	- mFFX - Gemcitabine + nab-paclitaxel	Basal-like subtype—worse prognosis on mFFX
Singh et al. [24]	2024	Palliative	- FFX - Gemcitabine + nab paclitaxel	Strongly basal-like subtype—worse prognosis, regardless of CTH regimen
O’Kane et al. [34]	2021		- mFFX	Classical subtype—favorable mFFX effect Basal-like subtype—little effect of mFFX

**Table 4:** Summary of main similarities and differences between molecular subtypes classifications of PDAC (QM-PDA—quasi mesenchymal pancreatic ductal adenocarcinoma; ADEX—aberrantly differentiated endocrine exocrine subtype).

Classification	Method Used	Stromal Cells Influence	Subtypes Determined	Similarities Between Classifications	Differences Between Classifications
Collisson [18]	hybridization array-based mRNA Non-negative matrix factorization	epithelium was microdissected away from the stroma (lack of stromal cells influence)	classical, QM-PDA, exocrine-like	QM-PDA overlapping with squamous and basal-like subtypes–due to loss of endodermal identity (poor prognosis) exocrine-like might be overlapping with ADEX subtype	lack of immunogenic subtype–due to lack of stroma cells undergoing analysis (microdissected).
Moffitt [19]	mRNA expression microarray and RNAseq Non-negative matrix factorization	stromal transcriptome subtracted from epithelial subtypes in analysis	basal-like and classical, and also described two stromal subtypes—normal and activated	basal-like overlapping with squamous and QM-PDA subtypes–due to loss of endodermal identity (poor prognosis) activated stroma corresponding to immunogenic subtype present (due to infiltration of immunogenic cells in stroma)	
Bailey [20]	mRNA expression microarray and RNAseq	full range of cellularity, including high epithelial content (>40%)	squamous, pancreatic progenitor, immunogenic, ADEX	squamous overlapping with basal-like and QM-PDA subtypes–downregulation of endodermal genes (poor prognosis) ADEX might be overlapping with exocrine-like subtype	immunogenic subtype present (due to infiltration of immunogenic cells)
Puleo [31]	RNA expression array	full range of cellularity	pure classical, immune classical, pure basal-like, stroma activated and desmoplastic	in high cellularity basal-like overlapping with squamous, basal-like and QM-PDA	immune classical subtype (associated with infiltration of immunogenic cells)

## Data Availability

The data presented in this study are available on request from the corresponding author.

## Supplementary Materials

The following supporting information can be downloaded at https://www.mdpi.com/article/10.3390/cimb48050502/s1.

## Abbreviations

The following abbreviations are used in this manuscript:

ADEX	Aberrantly differentiated endocrine exocrine pancreatic cancer subtype
CRT	Chemoradiation
DFS	Disease-free survival
DSS	Disease-specific survival
ECM-rich	Extracellular matrix-associated pathways subtype of pancreatic cancer
EGFR	Epithelial growth factor receptor
EMT	Epithelial–mesenchymal transition
FFPE	Formalin-fixed paraffin embedded
FOLFIRINOX/FFX	Chemotherapy regimen: 5-fluorouracil, irinotecan, oxaliplatin
GemCAP	Gemcitabine-capecitabine chemotherapy regimen
KRT17	Keratin 17
LAPC	Locally advanced pancreatic cancer
LCM	Laser capture microdissection
mFOLFIRINOX/mFFX	Modified FOLFIRINOX chemotherapy regimen
nab-paclitaxel	Nanoparticle albumin-bound paclitaxel
NAT	Neoadjuvant treatment
NMF	Non-negative matrix factorization
OS	Overall survival
PC	Pancreatic cancer
PDAC	Pancreatic ductal adenocarcinoma
PurIST	Purity-Independent Subtyping of Tumors
QM-PDA	Quasi-mesenchymal–pancreatic ductal adenocarcinoma
*RAS*	“Rat sarcoma virus” group of genes
RNA	Ribonucleic acid
RNAseq	Ribonucleic acid sequencing
SB	Strongly basal-like subtype of pancreatic cancer
SC	Strongly classical subtype of pancreatic cancer
TCGA	The Cancer Genome Atlas Research Network
