# Supplementary material for: Molecular Subtypes of Pancreatic Cancer: A Review of the Literature
Source: Curr Issues Mol Biol. 2026 May 13;48(5):502. doi: 10.3390/cimb48050502 (PMC13206680; doi:10.3390/cimb48050502)
Supplement: Supplementary file 1 [file cimb-48-00502-s001.zip › cimb-4250643-supplementary/cimb-4250643-supplementary.pdf]

Table S1. Characteristics of the included studies (OS—overall survival, QM-PDA—quasi-mesenchymal pancreatic ductal adenocarcinoma, FFPE—formalin-fixed paraf-fin-embedded, NMF—nonnegative matrix factorization, DFS—disease-free survival, DSS—disease-specific survival).

| Study | Year | Number of patients with molecular subtyping and survival analysis | Samples analyzed | Molecular subtyping method | Clinical stage | Prognostic outcome measure | Prognostic outcome (univariate analysis) | Prognostic outcome (multivariate analysis) |
|---|---|---|---|---|---|---|---|---|
| Collisson et al. [18] | 2011 | 27 | Resected tumor material, microdissected | Global gene expression analysis | Resectable tumor, cell lines | OS | Classical subtype tumors had better OS than QM-PDA subtype(p=0.038, log rank) | Molecular subtype was independent predictor of overall survival (p=0.024 Cox) |
| Moffitt et al. [19] | 2015 | 125 | FFPE (NMF clustering) | RNA sequencing | Resectable tumor | OS | 1)Basal-like subtype tumors had worse OS than classical subtype tumors (p=0.007)<br><br>2) Activated stroma subtype had worse OS than normal stroma subtype | NA |
| Bailey et al. [20] | 2016 | 96 | Resected tumor material | RNA sequencing | Resectable tumor | OS | Squamous subtype had worse OS | Squamous subtype had worse OS compared to other subtypes (HR: 2.23 (1.23 – 4.07); p = 0.0086) |
| Janky et al. [29] | 2016 | 118 | Resected tumor material (NMF clustering) | Gene expression profiles | Resectable tumor, metastatic PDAC | DFS and OS | 1) No statistically significant difference in OS between k2.cl1and k2.cl2<br><br>2)No statistically significant difference between the 3 subtypes separately (p = 0.193)<br><br>3) DFS better for k2.cl1 compared to k2.cl2 (p = 0.035)<br><br>4) DFS better for k3.cl1compared to k3.cl2 (p = 0.026) | Independent prognostic factors k2cl1 (HR 0.252 CI(0.092-0.888) p=0.034 |
| Birnbaum et al.[28] | 2017 | 601 | Resected tumor material, microdissected | Gene expression profiles | Resectable tumor | OS | 1) Moffitt: classical subtype had better OS than the basal-like subtype (p = 8.83E-07)<br>2) Bailey: squamous subtype had worse OS (p = 5.78E-08 )<br><br>3)Collisson: quasi-mesenchymal had worse OS (p = 1.65E-03) | In multivariate analysis, the Bailey's classification and Moffitt's classification remained significant (p = 1.69E-02 and p = 1.56E-03, respectively) |
| Puleo et al. [31] | 2018 | 288 | FFPE | RNA sequencing | Resectable tumor | DFS and OS | 1)Basal-like subtype had the worst outcome<br><br>2) Pure classical and immune classical subtypes showed an equivalently good log-rank test, p = 4 × 10–9) | In a multivariate model, the subtypes remained significantly associated with survival |
| Roa-Peña et al. [32] | 2019 | 124 | FFPE | RNA sequencing | Resectable tumor, metastatic PDAC | OS | Basal-like subtype was associated with worse prognosis (HR 1.799 p=0.0094) | NA |
| Maurer et al. [33] | 2019 | 60 | Fresh-frozen tumor tissue | RNA sequencing | Resectable tumor | OS | 1) Based on KM plot analysis basal-like had worse prognosis | NA |

| | | | | | | | | |
|---|---|---|---|---|---|---|---|---|
| | | | | | | | 2)Stromal subtypes are statistically associated with outcome in the ICGC cohort,with ECM-rich tumors having a worse prognosis | |
| O'Kane et al. [34] | 2021 | 195 | Fresh-frozen tumor tissue which undergoes laser capture microdissection | RNA sequencing | Resectable tumor, metastatic tumor | OS | OS in classical subtype was 9.3 months versus 5.9 months for basal-like PDAC (HR, 0.47; p = 0.0001). | In a multivariate Cox proportional hazard regression analysis, the Moffitt subtype remained highly prognostic (p = 0.018 |
| Suurmeijer et al.[30] | 2022 | 199 | Fresh-frozen tumor tissue | RNA sequencing | Resectable tumor | OS | Basal-like subtype had worse prognosis (HR 1.49, CI: 1.03 to 2.15; p = 0.035) | 1)Basal-like subtype was predictor for poor OS (HR 1.61, CI: 1.11 to 2.34; p = 0.013). 2)With histopathological variables in model, the prognostic value of subtyping decreased (HR 1.49, CI: 1.01 to 2.19; p = 0.045) |
| Dreyer et al. [26] | 2023 | 442 | Fresh-frozen tumor tissue | RNA sequencing and microarray gene expression | Resectable tumor | DSS | Squamous subtype was associated with significantly worse DSS ( p < 0.001) | Squamous molecular subtype was independently associated with worse prognosis [HR, 1.54 (CI: 1.042.28), p = 0.032) |
| Singh et al. [24] | 2024 | 3697 | FFPE | RNA sequencing | Metastatic PDAC | OS | Strongly basal-like subtype had worse OS | NA |
| Knox et al. [27] | 2025 | 253 | Biopsy after laser capture microdissection | RNA sequencing | Unresectable tumor, metastatic PDAC) | OS | NA | Basal-like subtype was associated with worse prognosis (HR 2.213, CI 1.522 -3.21; p<0.001) |
| Zhao et al. [35] | 2021 | 359 | FFPE | RNA sequencing and microarray gene expression | Resectable PDAC | DFS and OS | 1) Pure basal-like subtype and the stroma activated subtype showed the earliest relapse | NA |

Table S2. Molecular subtypes and their influence on treatment outcomes (NAT—neoadjuvant treatment, GemCAP—gemcitabine-capecitabine, mFFX—modified FOLFIRINOX)

| **Study** | **Year** | **Clinical stage** | **Number of patients** | **Treatment (adjuvant/ neoadjuvant/ palliative)** | **Regimens** | **Treatment outcomes** |
|---|---|---|---|---|---|---|
| Dreyer et al. [26] | 2023 | Resectable tumor | 442 | NAT and adjuvant | NAT:<br>-modified FOLFIRINOX<br>-gemcitabine -chemoradiation (CRT) with 50.4 Gy and GemCAP<br>Adjuvant:<br>decision of the treating oncologist | 1)Squamous subtype was less likely to receive adjuvant therapy.<br><br>2) Squamous subtype had worse prognosis, irrespective of whether adjuvant therapy was administered or not. |
| Collisson et al. [18] | 2011 | Human PDAC cell lines | NA | NA | NA | 1)QM-PDA subtype was more sensitive to gemcitabine.<br><br>2) Erlotinib was more effective in classical |

| | | | | | | |
|---|---|---|---|---|---|---|
| | | | | | | subtype cell lines. |
| Knox et al. [27] | 2025 | Unresectable disease | 253 | Palliative | Modified FOLFIRNOX (mFFX) or gemcitabine+nab-paclitaxel | Basal-like tumors demonstrated particularly poor OS when treated with mFFX (median OS 6.5 months, 95% CI 4.6–10). |
| Singh et al. [24] | 2024 | Metastatic disease | Gemcitabine and nab-paclitaxel: SB tumors (n = 112) and SC tumors (n = 178) FFX : SB tumors (n = 155) and SC tumors (n=215) | Palliative | FFX and gemcitabine + nab paclitaxel | Strongly basal-like tumors had significantly shorter OS compared to those with strongly classical tumors, regardless of first-line (1L) chemotherapy regimen. |
| O'Kane et al. [34] | 2021 | Resectable tumor and metastatic disease | 157 with available response rate data | - | mFFX | Favorable impact of mFFX in classical PDAC and little impact of mFFX in the basal-like population. |
